# Semantic segmentation of cerebrospinal fluid and brain volume with a convolutional neural network in pediatric hydrocephalus—transfer learning from existing algorithms

**DOI:** 10.1007/s00701-020-04447-x

**Published:** 2020-06-25

**Authors:** Florian Grimm, Florian Edl, Susanne R. Kerscher, Kay Nieselt, Isabel Gugel, Martin U. Schuhmann

**Affiliations:** 1 Department of Neurosurgery, University Hospital Tübingen, Hoppe-Seyler-Strasse 3, 72076 Tubingen, Germany; 2 Division of Pediatric Neurosurgery, University Hospital Tübingen, Tubingen, Germany; 3 Integrative Transcriptomics, Interfaculty Institute for Biomedical Informatics, University of Tübingen, Tubingen, Germany

**Keywords:** Deep learning, Image segmentation, CSF volume, Brain volume, Pediatric hydrocephalus, Area determination

## Abstract

**Background:**

For the segmentation of medical imaging data, a multitude of precise but very specific algorithms exist. In previous studies, we investigated the possibility of segmenting MRI data to determine cerebrospinal fluid and brain volume using a classical machine learning algorithm. It demonstrated good clinical usability and a very accurate correlation of the volumes to the single area determination in a reproducible axial layer. This study aims to investigate whether these established segmentation algorithms can be transferred to new, more generalizable deep learning algorithms employing an extended transfer learning procedure and whether medically meaningful segmentation is possible.

**Methods:**

Ninety-five routinely performed true FISP MRI sequences were retrospectively analyzed in 43 patients with pediatric hydrocephalus. Using a freely available and clinically established segmentation algorithm based on a hidden Markov random field model, four classes of segmentation (brain, cerebrospinal fluid (CSF), background, and tissue) were generated. Fifty-nine randomly selected data sets (10,432 slices) were used as a training data set. Images were augmented for contrast, brightness, and random left/right and X/Y translation. A convolutional neural network (CNN) for semantic image segmentation composed of an encoder and corresponding decoder subnetwork was set up. The network was pre-initialized with layers and weights from a pre-trained VGG 16 model. Following the network was trained with the labeled image data set. A validation data set of 18 scans (3289 slices) was used to monitor the performance as the deep CNN trained. The classification results were tested on 18 randomly allocated labeled data sets (3319 slices) and on a T2-weighted BrainWeb data set with known ground truth.

**Results:**

The segmentation of clinical test data provided reliable results (global accuracy 0.90, Dice coefficient 0.86), while the CNN segmentation of data from the BrainWeb data set showed comparable results (global accuracy 0.89, Dice coefficient 0.84). The segmentation of the BrainWeb data set with the classical FAST algorithm produced consistent findings (global accuracy 0.90, Dice coefficient 0.87). Likewise, the area development of brain and CSF in the long-term clinical course of three patients was presented.

**Conclusion:**

Using the presented methods, we showed that conventional segmentation algorithms can be transferred to new advances in deep learning with comparable accuracy, generating a large number of training data sets with relatively little effort. A clinically meaningful segmentation possibility was demonstrated.

## Background

The analysis of medical image data sets with the help of deep learning algorithms can be of great benefit for extended patient care and specialized diagnostics. Today, these algorithms can provide a solid foundation for segmenting and categorizing image data of any modalities [14, 22, 31].

In principle, deep-learning procedures are inspired by the anatomy of biologically occurring neural networks. The networks are composed of artificial neurons that are organized in multiple layers and that are connected between consecutive layers. Each neuron receives, weighs, and processes incoming signals and passes them on to further neurons in the next layer. The transmission of stimuli between artificial neurons is modeled mathematically. As in the biological model, the transmission of stimuli is characterized by the input signals (excitation and inhibition) and by the connection strength (weights) to the neurons of the deeper layers [21, 29].

The signal processing starts with the input layer, which receives external signals and which is passed on to an output layer via several hidden layers. In the output layer, the final classification results. The data processing within the network takes place in an increasing abstraction of the input signal. In the case of MRI recognition and classification, the input signal could be one MRI slide. In the first layers, for example, the recognition of surfaces and edges takes place; in the following layers, the compositions of shapes are recognized; and finally, possibly using further (deeper) layers, the input image is classified based on the recognition of individual features [3, 21].

The detailed structure and architecture of artificial neural networks vary greatly, and different procedures have been established depending on the area of application [14]. Today, convolutional neural networks (CNNs) are the most common and established standard for processing image data. These particular subspecies are inspired by the principle of the receptive field in biological signal processing of visual signals. Similar to the biological model, individual neighboring artificial neurons react to overlapping parts of the upstream visual field [9]. CNNs are very memory-efficient and provide comparably robust image recognition.

Compared with biological models, the learning mechanism of artificial neural networks is highly variable and comparatively ineffective. Unlike the biological model, standard networks do not learn during use but have to be trained with separate data in the first step to perform a classification task. Once trained, the network then remains static in further applications but solves classification tasks extremely fast. In most cases, supervised learning takes place when known data is transferred to the neural network. For learning, a mathematical method, backpropagation, is usually used: many pre-segmented or classified images are needed to train a neural network. First, the known input images are sequentially fed into the untrained network and classification is calculated. In comparison with the known basic truth, the classification error is calculated from this. In the following, the weights of the neuron connections are then slowly adjusted based on the error rate to approach the desired classification. In this way, a lot of known training data is required and the data is repeated cyclically and a respective computing time or powerful hardware is needed. The advantage is that the weights are adjusted independently of user interaction until sufficient classification accuracy is achieved [21, 29].

However, the data set must be large enough so that the network does not adapt too much to the training data set. If there is not enough data present during training, the training data set is well recognized, but the sensitivity decreases during the classification of unknown data in later use (overfitting) [34]. Overfitting can also occur if the desired modality, sequence, or organ system do not have a sufficient number of data sets. To increase the data to a certain amount, augmentation is the method of choice, for example by changing the size, rotation, or position of the training image data randomly.

Providing that sufficient amount of data for training is a considerable challenge. There is a huge amount of available medical imaging data in archives; however, this is not classified and therefore not available for training. It is particularly advantageous for segmentation if large amounts of available data are labeled with known ground truth. Each pixel or voxel of the data set indicates which class of organ or tissue it represents [14]. As this classification is typically done manually, it is an extremely time-consuming and expensive method. Therefore, already publicly available, pre-segmented training data sets are often used, which are only available in limited numbers and densities.

To overcome this issue and to generate large densities of labeled data, classical established algorithms could be part of the solution. Classical algorithms developed prior to the era of deep learning provide valid segmentation by filtering algorithms or individually adapting machine learning algorithms to address very specific questions, such as the segmentation of the human brain [25]. While these algorithms offer remarkable results for specific issues, they are often not clinically established due to their high technical complexity and the specificity of the problem. For general brain segmentation, several different algorithms already exist, but all of which fail to have a significant clinical implementation. The possibilities consist of semi-automatic segmentation [27], atlas-based [5] to extended algorithms with classical machine learning procedures [35, 39, 41]. Nevertheless, the implementation of volumetric analysis of the cerebral compartments brain and CSF also characterize an extended technical challenge as most of the algorithms are neither readily available nor free to use.

In our previous studies [10, 11], we addressed the segmentation of MRI data from children with hydrocephalus as a specific issue. Thin-layer true FISP data sets were used here, which in a T2-weighted approach provided reliable information on the anatomy and the amount of CSF [30]. Especially in the case of early childhood hydrocephalus, it is important to assess both the amount of cerebrospinal fluid and the brain volume as this crucial time of brain development determines the outcome [23, 24]. The CSF quantity yields information as to whether or not a therapy such as the implantation of a ventriculoperitoneal (VP) shunt or an endoscopic third ventriculostomy (ETV) may be successful. The development process of the brain volume, on the other hand, is more important for cognitive development and, thus, the outcome of the patient. Therapy of childhood hydrocephalus and its assessment should, therefore, be directed towards influencing the best neurocognitive outcome and thus should not only be assessed on ventricular size or CSF volume but also with information on brain volume.

For this reason, we implemented a well-known and widely used algorithm (FAST [42], FSL FMRIB Software Library [15]) for segmenting 3D data sets of hydrocephalic patients. FAST uses a robust segmentation algorithm based on a hidden Markov random field model, taking spatial orientation into account. The algorithm has already been implemented and adjusted into clinical practice and offers a reliable segmentation [25]. We could demonstrate that changes under therapy in brain volume and CSF can be reliably estimated automatically with this algorithm [10]. For this approach, however, the existence of the complete data set is required. For this reason, we investigated in the next step whether the area of CSF and brain on a representative 2D axial slice in the middle of the brain including the Foramen Monro would be sufficient. A very good correlation of volume and area was found so that an estimation of the clinical course is possible based solely on a single 2D layer [11].

Through this previous work [10, 12], a large amount of pre-segmented data was generated using the FAST algorithm. This study investigates whether a pre-initialized CNN (VGG16) can be trained in an extended transfer learning process with the generated segmented data and reliably deliver segmentation results. Furthermore, this paper examines whether the algorithm is capable of producing suitable segmentation results with known data. To do this, the BrainWeb data set [20] containing an artificially generated T2 data set with known ground truth is used. In the final step, it is evaluated whether the course of therapy can be assessed in clinical examples, as in the preliminary work on the segmentation of a single layer.

## Methods

### Study cohort

Ninety-five routinely performed true FISP MRI sequences (1 mm isovoxel) were retrospectively analyzed in 47 patients with pediatric hydrocephalus (male *n* = 24, mean 5.8 ± 5.4 years, posthemorrhagic hydrocephalus *n* = 14, obstructive hydrocephalus *n* = 30, postmeningitic hydrocephalus n = 1, external hydrocephalus n = 2). Postoperative imaging was included of *n* = 20 patients following a ventriculoperitoneal shunt (VP shunt) and *n* = 12 patients after endoscopic third ventriculostomy (ETV).

### Ground image segmentation

As performed in the previous studies [10, 12], a total of 95 routinely performed MRIs were evaluated using the freely available FMRIB Software Library (FSL). After preprocessing, the 3D data sets were fed into an automated script-based processing pipeline, consisting of the following steps: The first step was the masking of the inner skull compartments with the Brain Extraction Tool (BET) [33]. Subsequently, a 2-class segmentation into brain matter and CSF was carried out with FAST [42] with the result of 3-dimensional masks for the individual compartments. The segmentation of the remaining classes for tissue and background was performed using a threshold value (initially 30 units). If necessary, the threshold value was adjusted manually.

Each data set was visually inspected after segmentation to ensure a proper segmentation.

### Training and test data sets

Data analysis was performed with Matlab Deep Learning Toolbox (MATLAB, (2019), version 9.5.0 (R2019b), Natick, Massachusetts: The MathWorks Inc.)

Axial, anterior-posterior oriented sections were generated from the 3D MRT files and stored as geometry corrected image files with a 1-mm voxel resolution (256 × 180). With the previous segmentation, appropriately labeled image data were created with four classes (CSF, brain, tissue, background). Since some of the structural data sets also included neck tissue, basal, or head incisions without relevant tissue apically, only axial layers that showed brain tissue in the segmentation were selected. The complete data set was randomly split case wise so that 60% of the images (10,432 slices) were used as training data, 20% as validation data (3319 slices), and 20% test data (3289 slices). Since contrast and brightness values for MRIs differ significantly, image augmentation for these values was performed. Additionally, to the native training image, four images with the combinations of enhanced (+ 100%) or decreased (− 50%) contrast level and enhanced (+ 30%) or decreased (− 30%) brightness level were created. Additionally, random left/right translation and random X/Y translation of ± 10 pixels were used for data augmentation.

As the data set is pre-segmented data by the FAST algorithm with residual uncertainty, an additional test data set with known ground truth was created. For this purpose, the T2-weighted data set of the BrainWeb data set [20] (slice thickness 1 mm, noise 3%, intensity non-uniformity 20%) was used. The labels were taken from the available Anatomical Model of Normal Brain according to the above requirements and a corresponding labeled data set was generated. This resulted in an additional 111 labeled test images with known ground truth.

### Training of the segmentation network

A convolutional neural network (CNN) for semantic image segmentation composed of an encoder and corresponding decoder subnetwork was set up [2]. The network was pre-initialized with layers and weights from a pre-trained VGG 16 model [32].

The network used a pixel classification layer to predict the categorical label for every pixel in the input images. Class frequency of CSF (8.6%), brain (22.1%), tissue (14.3%), and background (55.0%) was obtained. Since the class “CSF” was underrepresented in the training data, a class weighting was carried out to balance classes.

A stochastic gradient descent with momentum (0.9) optimizer was used and a regularization term for the weights to the loss function was added with a weight decay of 0.0005. Cross-entropy was used as a loss function for optimizing the classification model. The initial learning rate was set to 0.001. Furthermore, the learning rate was reduced by a factor of 0.3 every 10 epochs. The network was tested against the validation data set every epoch to stop training when the validation accuracy converged. This prevented the network from overfitting on the training data set. The training was conducted on a single GPU (NVIDIA GeForce GTX 1060). The validation accuracy converged after 6000 repetitions.

### Validation of segmentation

The accuracy of the segmentation of the neural network was evaluated by segmenting the deferred test data and the BrainWeb data set. Accuracy scores and exemplary segmentation results are illustrated for both groups.

For comparability of the segmentation results, the following scores were calculated:

Accuracy as the ratio of correctly classified pixels to the total number of pixels.

$$ \mathrm{Accuracy}=\frac{\mathrm{TP}}{\mathrm{TP}+\mathrm{FN}}\kern0.5em \mathrm{TP}\hbox{---} \mathrm{true}\ \mathrm{positive},\mathrm{FN}\hbox{---} \mathrm{false}\ \mathrm{negative} $$

The boundary contour matching score (Mean BFScore) indicates how well the predicted boundary of each class matches the true boundary, defined as the harmonic mean of precision (Pc) and recall (Rc).

$$ \mathrm{Mean}\ \mathrm{BFScore}=\frac{2\ \mathrm{Pc}\ \mathrm{Rc}}{\mathrm{Pc}+\mathrm{Rc}} $$

The Intersection over Union (IoU, Jaccard similarity coefficient) indicating the amount of overlap per class.

$$ \mathrm{IoU}=\frac{\ \mathrm{TP}}{\mathrm{TP}+\mathrm{FP}+\mathrm{FN}}\kern0.5em \mathrm{FP}\hbox{---} \mathrm{false}\ \mathrm{positive} $$

And the commonly used Sørensen-Dice similarity coefficient.

$$ \mathrm{Dice}\ \mathrm{coefficient}=\frac{2\mathrm{TP}}{2\mathrm{TP}+\mathrm{FP}+\mathrm{FN}} $$

Additionally, confusion matrices were computed to illustrate the true and predicted classes for both data sets.

## Results

### Classification results test data

**Fig. 1:**
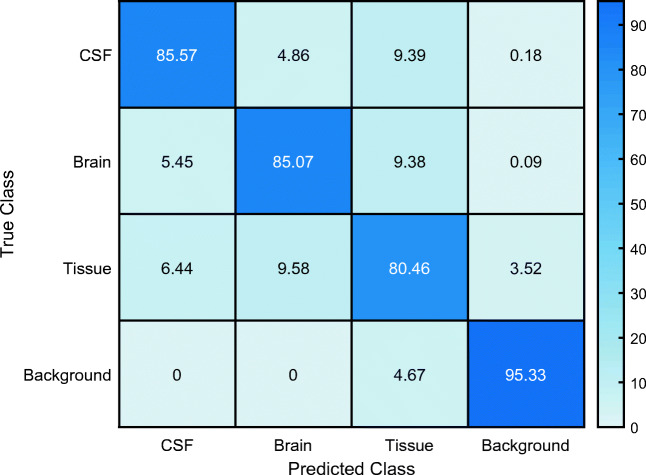
Confusion matrix of the segmentation result of the clinical test data. The columns represent the predicted class and the rows represent the true class. Data presented in % of classified pixels

**Fig. 2:**
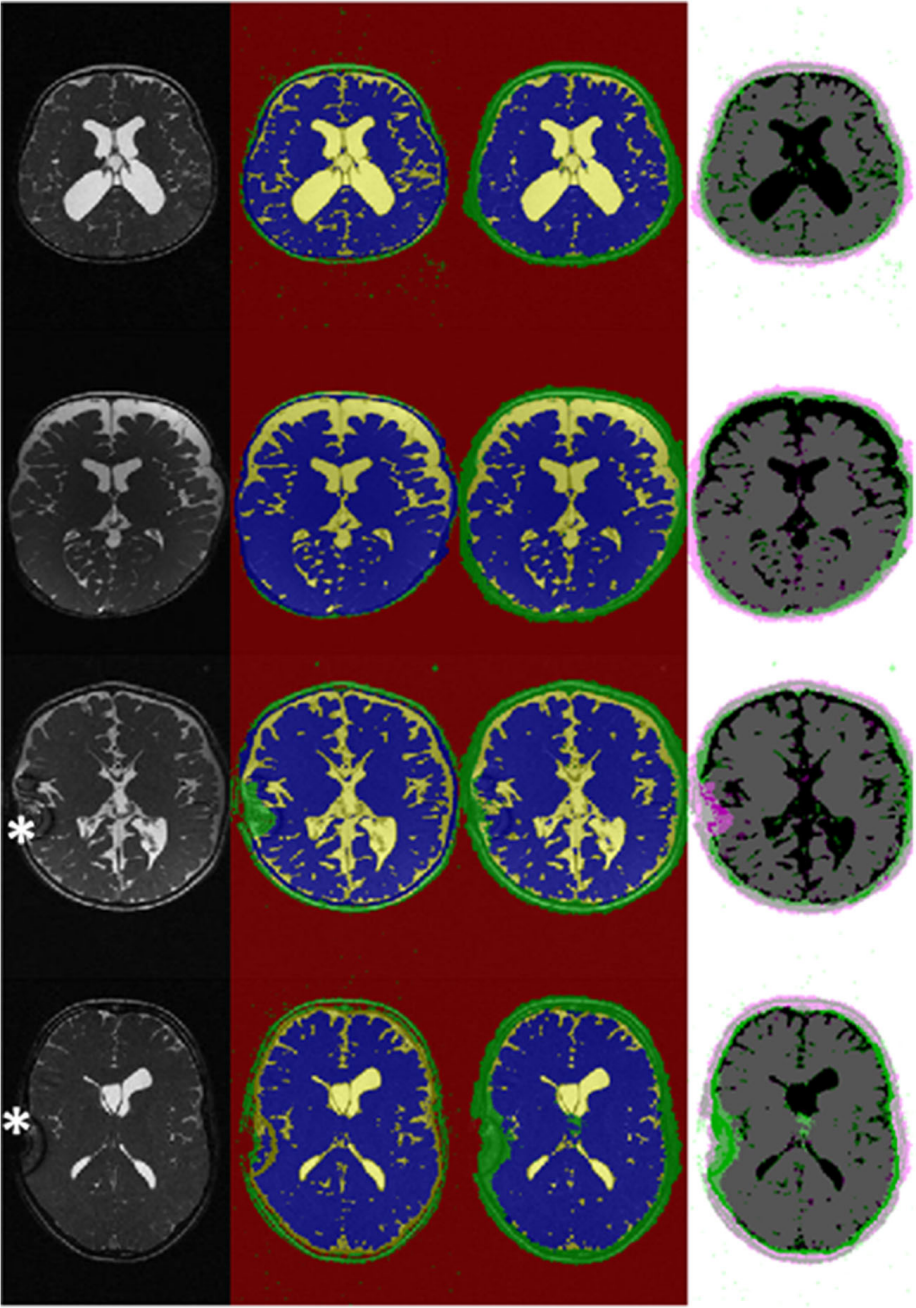
Segmentation examples of clinical test data set. From left to right: original T2-weighted true FISP images, ground truth segmentation (FAST, CSF yellow, brain blue, tissue green, background red), segmentation result of CNN, differences of segmentation (deviant classes in green and pink, concordant classes greyscale). From top to bottom: 1-year-old toddler with posthemorrhagic hydrocephalus, preoperative imaging; 9-month-old toddler with occlusive hydrocephalus, preoperative imaging; 3-year-old girl with occlusive hydrocephalus, control imaging 18 months postimplantation of a gravity compensated VP shunt; 12-year-old boy with occlusive hydrocephalus, 12 months after post implantation of a gravity compensated VP shunt. Particularly noteworthy are the susceptibility artifacts of the shunt valve in the patients of the last two rows (*) resulting in a false classification of FAST and CNN in this area

The classification results of the trained CNN were validated in 5046 randomly allocated test images (30% of the total data set). Table 1 and Fig. 1 give a detailed overview of the classification results as well as for the four classes’ brain, CSF, tissue, and background. Figure 2 shows exemplary segmentation results of individual patients with FAST and CNN.

**Table 1:** Classification results for global classification and each class separately of the clinical test data set. Accuracy, IoU (intersection-over-union), mean BFScore (boundary contour matching score), and Dice coefficient are reported for each class

	Accuracy	IoU	Mean BFScore	Dice coefficient
Global	0.90	0.74	0.83	0.86
CSF	0.86	0.68	0.70	0.81
Brain	0.85	0.79	0.79	0.88
Tissue	0.81	0.59	0.74	0.74
Background	0.95	0.94	0.84	0.97

### CNN segmentation results of the BrainWeb data set

**Fig. 3:**
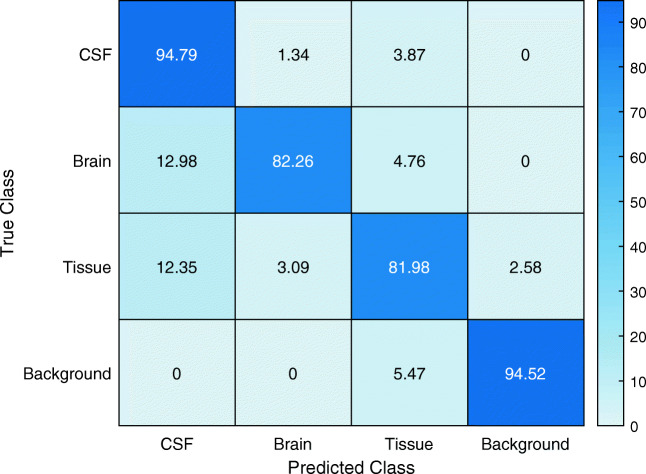
Confusion matrix of the CNN segmentation result of the BrainWeb data set. Columns represent the predicted class and rows the true class. Data presented in % of classified pixels

Additional validation of the segmentation was performed using the BrainWeb data set with known ground truth. This data set is a simulated T2-weighted data set. The segmentation results are shown in Table 2 and the confusion matrix in Fig. 3. Single layers of the BrainWeb data set with the segmentation results are shown in Fig. 5.

**Table 2:** Classification results of the CNN for global classification and each class separately of the BrainWeb data set

	Accuracy	IoU	Mean BFScore	Dice coefficient
Global	0.89	0.72	0.76	0.84
CSF	0.95	0.44	0.79	0.61
Brain	0.82	0.80	0.77	0.89
Tissue	0.82	0.71	0.57	0.83
Background	0.95	0.93	0.91	0.96

### FAST segmentation results of the BrainWeb data set

**Fig. 4:**
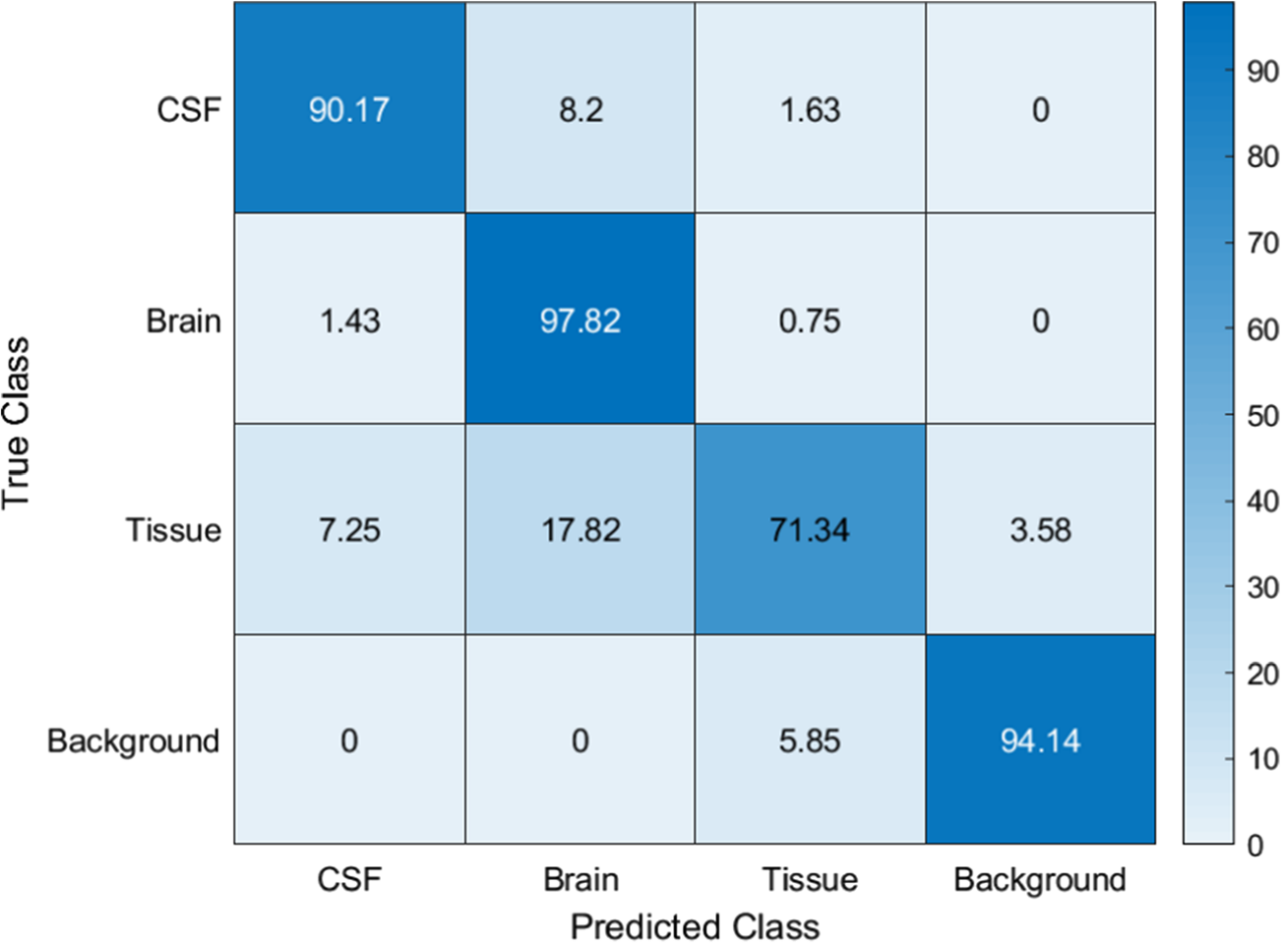
Confusion matrix of the FAST algorithm segmentation result of the BrainWeb data set. Columns represent the predicted class and rows the true class. Data presented in % of classified pixels

**Fig. 5:**
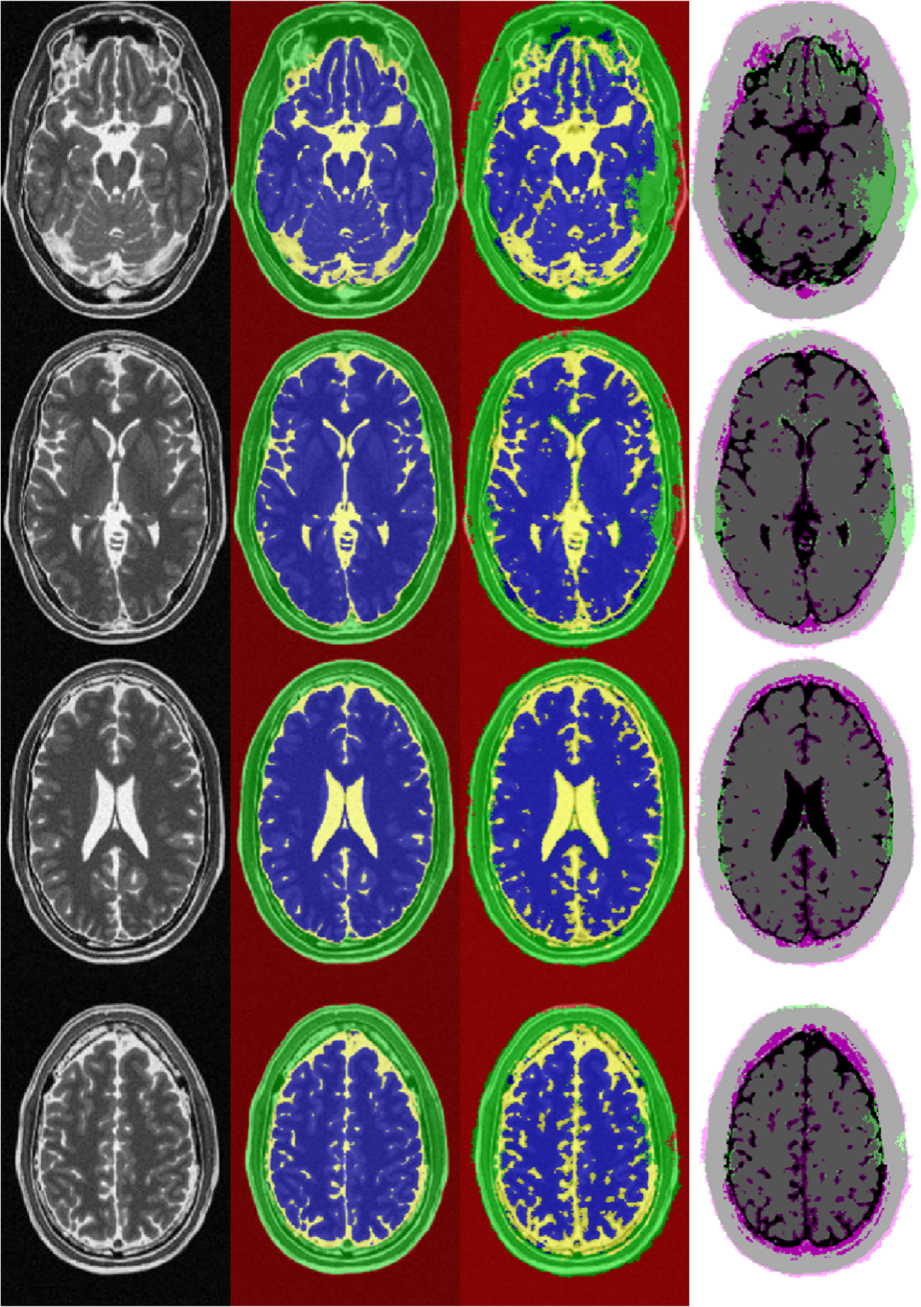
CNN and FAST segmentation examples of BrainWeb data set. From left to right: original T2-weighted images, ground truth segmentation (FAST, CSF yellow, brain blue, tissue green, background red), segmentation result of CNN, differences of segmentation (deviant classes in green and pink, concordant classes grey). From top to bottom: ascending slices of the same data set

For better comparability, the segmentation results of the FAST algorithm are also shown using the BrainWeb data. Table 3 and Fig. 4 display the validation data, while the segmentation of the individual layers is shown in Fig. 5.

**Table 3:** Classification results of the FAST algorithm for global classification and each class separately of the BrainWeb data set

	Accuracy	IoU	Mean BFScore	Dice coefficient
Global	0.90	0.77	0.73	0.87
CSF	0.90	0.67	0.88	0.80
Brain	0.98	0.85	0.73	0.92
Tissue	0.71	0.65	0.55	0.79
Background	0.94	0.92	0.76	0.96

### Therapy course examples

**Fig. 6:**
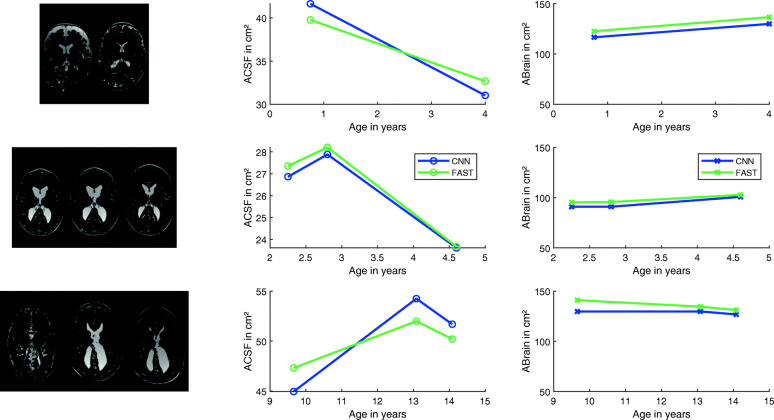
Exemplary clinical follow-up data. From left to right: original MRI-data on the level of the foramen of Monro, diagrams for ACSF and ABrain determined by CNN and FAST, diagrams for the estimated areas by CNN and FAST on the left. Each data point is represented by the MRI-images on the left. Top: implantation of a gravity-compensated VP shunt in an infant with occlusive hydrocephalus at the age of 9 months. The regular decrease in CSF volume and an increase in brain volume are reflected in area estimates in control imaging at the age of 4 years. Middle: primary VP shunt implantation in a 2-year-old boy with posthemorrhagic hydrocephalus, left is the pre-op scan. A slight increase of ventricular size was observed in the further course (second MRI from left). The adjustable valve unit was readjusted. In the subsequent MRI controls, a further decrease of CSF and an increase in brain mass occurred. Bottom: postoperative course imaging of a girl with occlusive hydrocephalus after early childhood implantation of a VP shunt. After an initial complication-free course with regular CSF drainage (initial picture at the age of 9 years on the left), there was an increasing underdrainage symptomatology with fatigue and headache. The subsequent control MRI showed a strong increase in ventricular space. Afterward, at the age of 13 years, operative shunt revision was performed with the immediate postoperative improvement of the symptoms. The control MRT examination showed a comparative decrease in the CSF spaces after revision

To demonstrate the clinical viability of the segmentation results, ACSF and ABrain are presented in selected childhood hydrocephalus patients with varying clinical courses. Figure 6 exhibits the underlying MRI slides at the level of the foramen of Monro as well as the area segmentation results of FAST and CNN.

## Discussion

In this work, we described how a classical, established machine learning algorithm can be conveyed to the modern method of segmentation using a pre-trained CNN in a double transfer learning algorithm. With existing specialized classical algorithms, it is relatively easy to create adequate training data sets with sufficiently accurate labeling. Moreover, it is possible to train a CNN with this large data set. The segmentation by the CNN provides valid results and is comparable with the segmentation results of the original algorithm. This can ensure valid segmentation with clinical data that is consistent with the results of the classical algorithm.

### Ground truth image segmentation to generate training data

The segmentation performance of the established FAST algorithm was transferred to our network by using its previously generated training data. Considering this ground truth in terms of reliability, the used FAST algorithm within the applied FSL-Toolbox [15] provided accurate segmentation results. FAST uses a robust segmentation algorithm based on a hidden Markov random field model, taking into account the spatial orientation of every voxel. The advantage of this algorithm is that segmentation can take place unaffected by the large anatomical variability often found in childhood hydrocephalus. Methods using atlas-based segmentation reach their limits in the case of severe anatomical changes, as the sometimes grotesquely altered ventricular cavities are often located far away from the conventional probability space. The FAST algorithm was evaluated in our previous studies in pediatric hydrocephalus. Each segmentation performed was reviewed and verified by medical professionals. An accurate segmentation for CSF and brain matter was found, and changes under therapy could be reliably assessed [10, 12].

In the MRBrainS Challenge [25], an external validation of the FAST algorithm took place. Three freely available segmentation toolboxes were evaluated (SPM 12 [25], Freesurfer [6], and FAST). FAST reached comparable good Dice coefficients of 93% for brain and 70% for CSF. The segmentation results of the MRBrainS Challenge are not directly commensurate, as the data sets used were T1, IR, and FLAIR sequences.

Due to the higher CSF contrast in the used modeled T2-sequence of the BrainWeb data set for internal validation, the FAST algorithm even performed better in our study with 80% Dice coefficient for CSF and comparing 92% for brain.

It appears therefore advantageous and appropriate to use existing algorithms to generate the labeled training data sets. The accuracy of the segmentation is clinically significant, and large amounts of data can be generated in a relatively short time. However, if the data must first be segmented manually, the costs and time involved are very considerable and less training data can be generated, leading to poorer segmentation results. Nevertheless, one can assume a very accurate ground truth by manual segmentation.

### Segmentation results of the CNN

As a comparable study in literature, Han et al. used manual segmentation of 600 data sets to determine CSF without brain in early childhood hydrocephalus [13]. The algorithm in this study achieved a Dice coefficient for CSF of 88%. In another recent study by Klimont et al., the segmentation was performed with a U-net using 85 CT data sets in pediatric hydrocephalus. The segmentation was likewise performed manually. Here, CSF without brain volume was considered. In this study, a Dice coefficient CSF of 95% could be achieved [18].

Overall, our segmentation results of the CNN on the BrainWeb data set were very accurate; however, the Dice coefficient cut off was marginally inferior due to slightly higher false negative values in the tissue area, particularly in the lower layers. As these areas are not included in the classification of the FAST algorithm due to the previous extraction of the brain by BET, the previous masking of the inner skull spaces appears to be advantageous here. Furthermore, in the classical T2 sequences, the tissue fat signal was overrepresented when compared with the underlying training data with true FISP sequences. The used true FISP sequences in our study are comparatively fast and offer high spatial resolution and excellent CSF contrast, making them therefore optimal for imaging pathologies of the CSF system [30].

The validation of the deferred clinical data sets shows optimal results with an overall accuracy of 90% and Dice coefficient for brain of 88% and CSF of 81%. The Mandell group achieved comparable values for brain (94%) and weaker results for CSF (57–67%) in the segmentation of pediatric hydrocephalus patients [24]. Han et al. achieved a Dice coefficient for CSF of 88%; a brain segmentation was not performed here [13]. With our method, clinically relevant areas or changes in volume can be detected. Figure 6 illustrates by the patient examples that the calculated areas can map an accurate course of CSF and brain mass making it possible to visualize clinically relevant changes.

### Network structure and transfer learning

In regard to the network structure and training data set in our study, we followed a multiple transfer learning approach. Transfer learning describes the ability of a system to use the knowledge acquired in a previous task for a new classification [4]. The study used a pre-initialized CNN designed for visual recognition (VGG 16), upon which the original weightings of the network were adopted and then fine-tuned with the training data. The closer the classification tasks of the networks are to each other, the better the classification accuracy can be achieved through transfer learning. Even if the original tasks are further apart, it could be shown that maintaining the weightings was more effective than randomizing the initialization of the network [34, 40]. This is similar to the studies with prenatal images by Wu et al. [37], who used the original weights from a general network (VGG 16) and then fine-tuned on the training data.

### 3D volumetry and 2D image segmentation

With the CNN segmentation algorithm described, both a two-dimensional (area determination) and a three-dimensional (volumetry) can be performed. As demonstrated in our previous work [10] and by other authors [23, 26, 36], 3D volumetry is an advanced method for quantitative and precise monitoring of the course of therapy in childhood hydrocephalus. Furthermore, only quantitative data on changes in brain and CSF provide an accurate foundation for comparing different treatment modalities. Previous volumetry studies have shown that the neurocognitive outcome depends primarily on the development of brain mass. In particular, the CSF volume may be less significant than the course of the brain volume when examining cognitive, motor, and speech development. It is postulated that these neurocognitive changes are caused by white matter lesions due to increased brain pressure and ventricular dilatation [7]. The volume effect of the brain mass would be a rather indirect sign to estimate these alterations. Therefore, the approach of determining the three-dimensional CSF and brain volume is preferable and a more objective basis for a comprehensive treatment evaluation [23, 24].

Howbeit, the volumetry method requires a complete 3D data set and significant computing time and power. The computing effort can be reduced through automation as previously shown [10]. Furthermore, patients with congenital hydrocephalus often require several MRI examinations to evaluate the course of their disease, especially concerning any necessary interventions or to check the functionality of an ETV or a VP shunt. Although MRI scans have no radiation exposure and are therefore preferred to CT scans for follow-up checks, significantly longer examination time is required. This poses a problem for younger children in particular as they often cannot lie still for the entire duration of the examination and the quality of the MRI images is therefore significantly limited by movement artifacts. For this reason, sedation during the examination is almost always necessary in children [28].

The proposed method of planar area determination eliminates some of these disadvantages. There is no need for a complete thin-sliced 3D data set as an artifact-free slice in the plane of the foramen of Monro would be sufficient. The area result correlates excellently with the volumes of the compartments, and faster non-3D sequences can be used as a basis for an area-based evaluation. For a follow-up examination, it would be conceivable to perform only a short sequence including the proposed reference plane of high quality. Due to the excellent correlation, a reliable prediction of CSF and brain volume seems to be possible from the area data [11].

### Limitations of the study

The clinically reliable calculation of the volume of an area determination in 2D resulted in the use of a 2D CNN as the basic network structure. Due to the excellent initial data situation with the available high-resolution 3D data sets and the already existing exact segmentation, a 3D CNN for the segmentation would also have been conceivable. Based on the higher spatial information in 3D, a greater accuracy of the segmentation could have been expected. While the benefits of 3D CNNs have been demonstrated in preliminary studies [8, 17], using a 3D model would have significantly influenced the clinical applicability of the method. As with the FAST algorithm, the complete 3D data set would have had to be available artifact-free and in digital form and the processing of the data would have required increased technical and time expenditure. The pre-trained 2D CNN, which was developed within the scope of this study, can be easily transferred to e.g. a smartphone app, which could be used for volume estimation via screenshot in the clinical routine.

A general limitation of the comparability of study results is that only a certain number of validated test data sets are available for different modalities. For example, there are pre-segmented test data sets of the MRBrainS Challenge [25] in the modalities FLAIR (fluid-attenuated inversion recovery), IR (inversion recovery), and T1, but not for T2-weighted images. Pre-segmented true FISP data sets are not available at all to our knowledge. The used BrainWeb data set consists of artificially simulated MRI data and was originally designed to validate various segmentation algorithms as a known basic truth [20]. It exhibits similarity of image morphological structures to in vivo acquired MRI data [20, 38] and is used frequently in studies for verification purposes [1, 16, 19].

With the proposed method, misclassifications were found in the area of susceptibility artifacts of the shunt implant since there was often complete signal annihilation. One disadvantage of our method is that systematic errors made by the underlying segmentation algorithm are learned by the CNN. An example of this can be seen in Fig. 2. Here, FAST and later CNN consistently incorrectly evaluate the susceptibility artifacts of the shunt system as tissue area. Of course, extinction artifacts pose a challenge to all algorithms, and possibilities of recognizing these artifacts and extrapolating them must be investigated in the future.

## Conclusion

Transfer learning from established classical segmentation algorithms via deep learning techniques is a promising method to establish a reliable segmentation. Large data sets can be created rather quickly, which are needed to achieve reliable segmentation accuracy through network training. With the created segmentation algorithm, MRI data can be segmented with reliable accuracy and the clinical course of brain and CSF development can be traced.

The presented area determination on a single layer allows an exact estimation of the volume of brain and CSF. These deep learning algorithms can be integrated into other applications e.g. via tensor flow. It would be logical to integrate the algorithms into a mobile phone app to allow broad access to this method. Thus, quantitative therapy monitoring in pediatric hydrocephalus therapy could be performed in daily practice and serve as a precise basis for future analysis and comparison of treatment options.

For a further generalization of the method, additional training of the network using other common imaging modalities is required. To expand this method to adult hydrocephalus, segmentation of CT data is necessary. For future research, it must be shown that an even more precise segmentation outside the intracranial spaces can be achieved, e.g. by additional probabilistic masks. Furthermore, it must be demonstrated how susceptibility artifacts can be extrapolated e.g. by mirroring the opposite side or by further deep-learning procedures.

## Abbreviations

CSF: Cerebrospinal fluid
VP shunt: Ventriculoperitoneal shunt
ETV: Endoscopic third ventriculostomy
MRI: Magnetic resonance imaging
ABrain: Planar area of brain in cm^2^
ACSF: Planar area of CSF in cm^2^
FSL: Functional Magnetic Resonance Imaging of the Brain Software Library
BET: Brain extraction tool
FAST: FMRIB’s Automated Segmentation Tool
true FISP: True fast imaging with steady state precession
SD: Standard deviation
TP: True positive
FN: False negative
FP: False positive
BFScore: Boundary contour matching score
IoU: Intersection-over-union
CNN: Convolutional neural network
